# Evolution of Research on Persistent Postural-Perceptual Dizziness: A Bibliometric and Visualization Analysis from 1994 to 2025

**DOI:** 10.3390/audiolres16020052

**Published:** 2026-04-01

**Authors:** Jiyu Zhang, Shuqi Yao

**Affiliations:** 1 Luoyang Central Hospital, Luoyang 471000, China; 20243169@snnu.edu.cn; 2 School of Sports, Shaanxi Normal University, Xi’an 710119, China

**Keywords:** persistent postural-perceptual dizziness, bibliometric analysis, vestibular migraine, vestibular rehabilitation, functional dizziness

## Abstract

**Background**: Persistent Postural-Perceptual Dizziness (PPPD) is a chronic vestibular disorder that has been receiving more research attention lately. Nonetheless, there is a lack of systematic bibliometric overviews tracing the conceptual evolution, knowledge structure, and emerging research frontiers within this field. The utilization of bibliometric and visualization analyses can enhance the understanding of trends and central themes in PPPD research, offering valuable insights for future studies. **Methods**: Data were retrieved from the Web of Science Core Collection, yielding a final dataset of 370 bibliographic records (“DATA”). We employed CiteSpace, HistCite, the Alluvial Generator, and R software to conduct multi-dimensional statistical and visualization analyses on publication trends, collaborative networks (countries/institutions/authors), disciplinary distribution, citation bursts, and the evolution of keyword clusters. **Results**: Starting from 2005, there has been a notable increase in publication volume, reaching its peak in 2024. The United States and Germany are at the forefront of national collaboration, with the University of Munich and the Mayo Clinic being key research institutions. The research focus has transitioned from a primary emphasis on Psychiatry to a broader scope encompassing Neurosciences, Otorhinolaryngology, and General Medicine. Keyword analysis reveals a shift towards standardized terminology, transitioning from “phobic postural vertigo” to “diagnostic criteria” and “consensus documents”. Current research trends are centered around comorbidity mechanisms like “vestibular migraine”, therapeutic approaches such as “vestibular rehabilitation”, and quality of life assessments using the “dizziness handicap inventory”. The 2017 consensus document by the Bárány Society is highlighted as a pivotal publication with significant citation impact. **Conclusions**: The intellectual structure of the field, as revealed by this bibliometric analysis, has transitioned from a phenomenological description to a conceptual unification. The bibliometric analysis indicates that the field is currently in a conceptually stabilized stage characterized by a research focus on refining diagnostic precision and comorbidity exploration, while scholarly attention remains biologically exploratory regarding objective biomarkers and pathophysiological mechanisms.

## 1. Introduction

Dizziness and vertigo constitute a massive global health burden, ranking among the most frequent complaints in both primary care and emergency departments, with a lifetime prevalence approaching 20–30% in the general population [1]. Among the spectrum of vestibular pathologies, chronic dizziness poses a particularly stubborn clinical challenge, often leading to prolonged disability, socioeconomic loss, and significant psychological distress [2]. Historically, patients presenting with chronic unsteadiness without overt structural deficits were frequently marginalized under ambiguous labels such as “psychogenic dizziness” or “phobic postural vertigo”, hindering the development of targeted therapeutic interventions [3]. This landscape was fundamentally transformed in 2017 when the Bárány Society formally defined Persistent Postural-Perceptual Dizziness (PPPD), unifying these precursor concepts into a distinct functional vestibular disorder [4].

Clinical studies identified in this analysis increasingly characterize PPPD as a common cause of chronic dizziness in middle-aged adults, manifesting as a fluctuating sense of unsteadiness and non-spinning vertigo that persists for months or years [5]. The disorder is characteristically exacerbated by upright posture, active or passive motion, and exposure to complex visual stimuli—a phenomenon termed “visual vertigo” [6]. Recent pathophysiological investigations have shifted the understanding of PPPD from a purely psychiatric etiology to a functional neurological framework. A growing body of literature focused on pathophysiology suggests a framework where PPPD arises from maladaptive neuroplasticity, where the brain fails to “readjust” after an acute vestibular event, such as vestibular neuritis or benign paroxysmal positional vertigo (BPPV), resulting in a persistent over-reliance on visual inputs for postural control [7]. Neuroimaging studies utilizing resting-state functional MRI (rs-fMRI) have further corroborated this, revealing altered functional connectivity in the vestibulo-visuo-somatosensory networks and spatial cognitive cortical areas [8,9].

The clinical complexity of PPPD is compounded by its high rates of comorbidity. A substantial proportion of patients exhibit overlapping features with Vestibular Migraine (VM), creating a diagnostic “grey zone” that complicates management [10]. Furthermore, the bidirectional relationship between vestibular dysfunction and anxiety disorders creates a vicious cycle; anxiety not only precipitates PPPD but also perpetuates the “high-risk” postural control strategies that maintain symptoms [11]. Consequently, the research landscape as mapped by our analysis has evolved toward a multimodal paradigm. The literature increasingly reports on randomized controlled trials and systematic reviews that explore the role of Vestibular Rehabilitation Therapy (VRT) [12,13] and Cognitive Behavioral Therapy (CBT) [14,15], often combined with serotonergic pharmacotherapy, to dismantle these maladaptive networks.

As the volume of literature expands rapidly—with preliminary data suggesting a peak in recent years—the ‘fog’ of information. The rapid proliferation of publications makes it increasingly difficult for clinicians and researchers to manually synthesize global trends and identify core intellectual shifts. While bibliometric analyses have successfully mapped the research trends of other neuro-otological conditions, such as BPPV [16], Meniere’s Disease [17], and Vestibular Schwannoma [18], a comprehensive, data-driven visualization of the PPPD research landscape remains notably absent. Traditional narrative reviews [19], while valuable for synthesizing clinical insights and pathophysiological theories, often lack the quantitative capacity to objectively disentangle complex global citation networks or trace the longitudinal structural evolution of the field. Unlike qualitative summaries, this study provides a high-resolution, data-driven “road map” of the PPPD research landscape from its inception to 2025. The unique value of our bibliometric approach lies in its ability to (i) identify “citation bursts” that signal pivotal shifts in scientific attention; (ii) map the global scientific collaboration architecture involving 537 institutions and 39 subject categories; and (iii) utilize alluvial flow diagrams to visualize the longitudinal “metamorphosis” of research themes from early phenomenological constructs to modern modular concepts. By decoding these patterns, this study offers an objective perspective on the field’s intellectual lineage that narrative methods cannot capture, thereby enabling a more precise forecasting of emerging research frontiers such as digital therapeutics and comorbidity-informed precision medicine.

To bridge this gap, this study employs advanced bibliometric tools—including CiteSpace, HistCite, and the Alluvial Generator—to conduct a multi-dimensional analysis of global PPPD research from 1994 to 2025. The search commenced in 1990 to ensure comprehensive coverage, while 1994 represents the publication year of the earliest identified study involving the precursor concept of phobic postural vertigo. By decoding the “intellectual lineage” from early phenomenological descriptions to the current era of precision medicine, we aim to provide researchers and clinicians with a high-resolution map of the field’s evolution. This analysis not only highlights the shifting disciplinary focus from Psychiatry to multidisciplinary Neuroscience and Otolaryngology but also identifies critical future hotspots, such as the mechanisms of visual dependence and the optimization of digital therapeutics [20,21].

## 2. Methods

### 2.1. Data Acquisition and Statistical Processing

This study utilized a Topic Search (TS) strategy to search the Web of Science Core Collection (WoSCC). To ensure the highest possible coverage and data quality, we included the following indices: Science Citation Index Expanded (SCI-EXPANDED), Social Sciences Citation Index (SSCI), and Emerging Sources Citation Index (ESCI). The retrieval timeframe was set from 1 January 1990 to 10 December 2025 (the final retrieval date).

The exact search string executed was as follows: TS = (“Persistent postural-perceptual dizziness” OR “Persistent postural perceptual dizziness” OR “Chronic Subjective Dizziness *” OR “Phobic Postural Vertigo *”). No language restrictions were applied during the initial search to prevent linguistic bias. The WoSCC was selected as the sole data source because it provides the most comprehensive and standardized citation metadata (including titles, abstracts, and all cited references), which is essential for the high-fidelity citation-based mapping and longitudinal tracking required by tools like CiteSpace and HistCite [22]. Although the search timeframe was initiated from 1990 to ensure no early literature was missed, the first identified record in this field appeared in 1994, which defines the effective analysis period (1994–2025) as stated in the title. The data screening and inclusion process followed a rigorous, standardized workflow to ensure reproducibility. First, the retrieval was limited to specific document types: only “Articles” and “Review Articles” were included. Other document types, such as meeting abstracts, editorial materials, letters, news items, and proceedings papers, were excluded as they often lack complete citation metadata or peer-reviewed empirical data. Second, a two-stage screening was performed: two authors (J.Z. and S.Y.) independently reviewed the titles and abstracts of all retrieved records. Duplicates were removed using the internal deduplication function of CiteSpace. Documents were excluded if they were deemed “irrelevant” (e.g., studies focused on dizziness as a side effect of unrelated pharmacological agents or structural ear pathologies that did not address the functional diagnostic criteria of PPPD). The authors finally obtained 370 relevant and academically valuable documents that met the research topic criteria, forming the final dataset (referred to as “DATA” hereafter). The cleaned and deduplicated dataset used for visualization is available in Supplementary Materials. The retrieved documents were exported in the format of “Full Record and Cited References” and saved as plain text files for further analysis. Ensuring the inclusion of complete metadata such as title, authors, institutions, keywords, abstracts, publication year, journal name, and references.

First, within ‘Time Slicing’, define the analysis period based on the literature’s temporal scope (e.g., 1994–2025), and set the time slice (Years Per Slice) to ‘1’ to enable annual analysis. Subsequently, under ‘Node Types’, select node classifications according to research objectives: utilize ‘Keyword’ to identify research hotspots, employ ‘Author’ or “Institution” to uncover core authors and collaboration networks, and conduct co-citation analysis via ‘Cited Reference’ to probe the knowledge base. For threshold settings, the g-index (e.g., k = 25) is typically employed to extract high-frequency nodes within each time slice. Under ‘Pruning’, select either “Pathfinder” or ‘Pruning sliced networks’ to simplify the graph structure and highlight critical pathways. Upon completing these configurations, run the software to generate visualizations for subsequent clustering analysis and evolutionary trajectory mining.

To ensure the accuracy and high quality of the data, the research team rigorously screened the search results, excluding duplicates, irrelevant, and non-academic literature using Microsoft Excel (WPS Office 2019 version) for data management and systematic relevance checks. The authors finally obtained 370 relevant and academically valuable documents that met the research topic criteria, forming the dataset (referred to as “DATA” hereafter). Based on this dataset, core information such as the publishing country/region, authors and their affiliations, journal sources, and types of literature (such as original research papers, review articles, etc.) was extracted and organized. Subsequently, descriptive statistical analysis was conducted using Microsoft Excel (WPS Office 2019 version), laying a solid foundation for further in-depth analysis.

### 2.2. Bibliometric Analysis Tools

#### 2.2.1. CiteSpace Analysis

Co-occurrence Networks: We employed CiteSpace to map the intellectual structure of the field. In the context of bibliometrics, a scientific partnership is operationally defined as the concurrent appearance of multiple authors, institutions, or countries/regions within the byline of a single publication. Given that scientific research necessitates extensive collaboration, the examination of these cooperative networks reveals the underlying research landscape of a specific scientific domain. When the dataset is imported into the CiteSpace software, these synergistic relationships and scientific concepts are visualized as a co-occurrence network. CiteSpace utilizes a color-coded visualization system for nodes and edges to delineate the merged network structure. Colors are assigned based on the year of publication within the dataset. The color of a network edge corresponds to the year in which the co-occurrence link was first established. Nodes are visualized as “tree rings,” composed of concentric circles of varying colors; the thickness of each ring is proportional to the frequency of co-occurrence in a given year. A red ring signifies a “citation burst,” indicating a sudden surge in citations during that specific year. Conversely, a purple ring denotes high “betweenness centrality.” Nodes possessing high betweenness centrality are statistically significant as they serve as pivotal bridges connecting different clusters or disparate nodes within the network.

Burst Detection: Building upon the work of Jon Kleinberg [23], who posited that streams of documents (such as emails or articles) exhibit specific thematic bursts that emerge and subsequently fade over time, we utilized specific text mining algorithms to identify these temporal thematic shifts. These shifts are characterized as “activity bursts.” Following the methodology of Chen et al. [24], which adapts Kleinberg’s algorithm, we defined a citation burst as a reliable indicator of an active topic. A citation burst detects events where attention to a specific topic increases sharply, lasting for a single year or persisting over multiple years. CiteSpace provides burst detection capabilities for subject categories, keywords, and references. The emergence of a citation burst serves as evidence that a particular discipline, keyword, or reference is associated with a surge in scholarly attention—in other words, it has triggered intense interest within the scientific community.

Cluster Analysis: CiteSpace offers three distinct clustering algorithms based on titles, abstracts, and keywords to categorize publications into conceptual clusters with defining research characteristics. Depending on the slicing settings, the cluster mapping reflects the evolution of conceptual clusters across different periods. Furthermore, timeline mapping provides a clear visualization of the genesis and decline of specific clusters, as well as the nodes associated with other clusters.

Specific Protocol: The “DATA” dataset concerning PPPD was imported into CiteSpace software (version 6.2.R4). The “Time Slicing” parameters were configured to cover the period from 1994 to 2025, with a slice length of one year per slice for micro-temporal burst detection. For each time slice, node selection was performed using the g-index algorithm with the scaling factor k set to 25. This criterion was chosen to ensure the inclusion of sufficiently representative nodes while maintaining network clarity. To generate the knowledge maps, we selected source terms including “Title,” “Abstract,” “Author Keywords (DE),” and “Keywords Plus.” Node types were selected according to the specific analysis (e.g., Country, Institution, or Author) while keeping other settings at their default values to automatically generate collaboration network knowledge maps. To optimize the network topology and eliminate redundant links, we applied the “Pathfinder” and “Pruning Sliced Networks” algorithms, which enhance the structural clarity of the resulting visualizations. These maps were subsequently manually adjusted to optimize clarity and esthetic presentation. A similar protocol was utilized to construct the keyword cluster map, with the distinction that “Keyword” was selected as the node type. Conceptual clustering was executed using the Log-Likelihood Ratio (LLR) algorithm. The quality and structural reliability of the generated clusters were evaluated using two metrics: Modularity (Q) and Mean Silhouette (S). In this study, our primary networks yielded Q values > 0.5 and S values > 0.7, indicating a robust community structure and high cluster homogeneity. The time slicing for keyword cluster snapshots was segmented into distinct phases: 1994–2005, 2006–2015, 2016–2020, and 2021–2025. These intervals were strategically selected to represent the field’s evolution from the early foundational decade (1994–2005) and the expansion phase (2006–2015), to the critical post-consensus period (2016–2020) and the most recent five years of frontier exploration (2021–2025). Additionally, to generate the citation timeline map, “Reference” was selected as the node type; within the “Control Panel,” we accessed the “Layout” tab and subsequently the “Timeline View” option. For burst detection (keywords, categories, and references), the “Burstness” parameters were configured with a minimum duration of 2 years and a gamma value of 1.0 to ensure that identified bursts represented significant and sustained shifts in research focus rather than transient fluctuations. Finally, by selecting the “Burstness” tab in the “Control Panel” and clicking “View,” we generated burst maps for keywords, categories, and references.

#### 2.2.2. HistCite Analysis

HistCite Pro (version 2.1) was utilized to construct historiographic diagrams and extract the most significant literature, enabling rapid identification of highly cited works. HistCite evaluates articles using two primary metrics: the Local Citation Score (LCS) and the Global Citation Score (GCS). The LCS refers to the frequency with which a study is cited within the analyzed collection (the software’s internal dataset), whereas the GCS refers to the total citation frequency within the entire Web of Science Core Collection database.

We imported the 370 research articles from the “DATA” dataset into HistCite Pro 2.1. To map the intellectual lineage of the PPPD research field, the “Limit” parameter was set to 30, with all other settings retained at default values. The “Graph Maker” function was then executed to visualize the citation chronologies and locate pivotal literature.

#### 2.2.3. The Alluvial Generator

Alluvial flow diagrams were constructed to elucidate temporal patterns within evolving networks. To generate these diagrams, we first utilized CiteSpace to produce a series of individual networks based on co-occurring keywords. These networks were exported from CiteSpace and subsequently loaded into the Alluvial Generator (available at http://www.mapequation.org/apps/AlluvialGenerator.html, accessed on 20 October 2025). In this visualization, each keyword is treated as a distinct node. Nodes are clustered within each time slice, and each cluster is conceptualized as a module. Across different time slices, nodes may split or merge to form new modules, with the most recent modules being formed by the intersection of preceding nodes, thereby illustrating the flow and evolution of scientific concepts over time.

#### 2.2.4. Statistical Visualization with R

The donut chart presented was constructed using the R statistical computing environment (version 4.2.2). The visualization was generated utilizing the geom_bar function within the ggplot2 package (version 3.4.4), a comprehensive system for declaratively creating graphics.

## 3. Results

### 3.1. Historical Characteristics of the Literature

#### 3.1.1. Distribution of Publications

The temporal evolution of scientific literature serves as a critical proxy for knowledge accumulation, offering quantitative insight into the developmental maturity of a research domain. As summarized in Table 1, the dataset comprises 370 publications across 146 journals, representing a multidisciplinary effort involving over a thousand authors and institutions. This descriptive overview provides the quantitative basis for interpreting subsequent visualizations, including growth phases of the field and the diversification of clinically relevant research themes.

**Figure 1:**
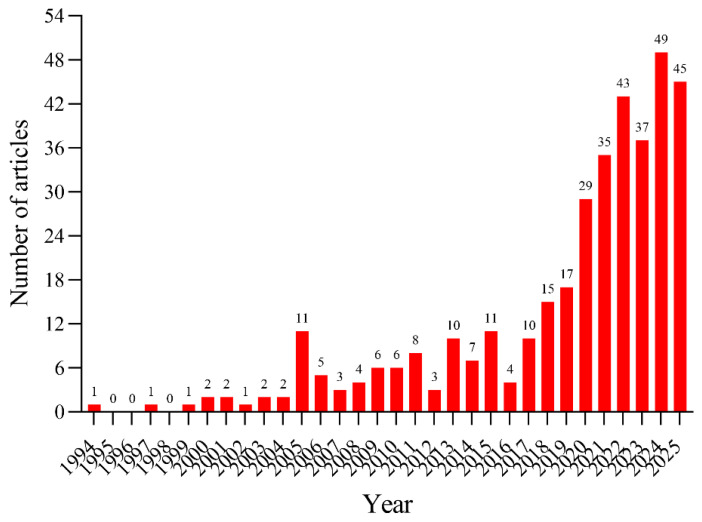
Annual distribution of publications on Persistent Postural-Perceptual Dizziness (PPPD), 1994–2025. The curve depicts the temporal growth of PPPD-related literature, showing an early low-output period followed by a sustained rise after 2005 and an accelerated expansion after the 2017 Bárány Society consensus criteria. The peak around 2024 reflects intensified research activity and broader clinical recognition of PPPD as a standardized diagnostic entity rather than a diagnosis of exclusion.

The trajectory of annual research output, visualized in Figure 1, reveals a distinct growth pattern. The field’s genesis (1994–2004) was characterized by an initial accumulation phase with low annual output. However, a pivotal shift occurred post-2005, marking the onset of a rapid expansion phase that continued through 2019. This upward momentum accelerated further in the subsequent years, culminating in a peak publication volume in 2024, signaling a field that is currently attracting intense scientific interest. Notably, the acceleration after 2017 temporally coincides with the publication of the Bárány Society diagnostic criteria, suggesting that conceptual standardization may have facilitated broader clinical uptake and stimulated research expansion across diagnosis, comorbidities, and management.

**Figure 2:**
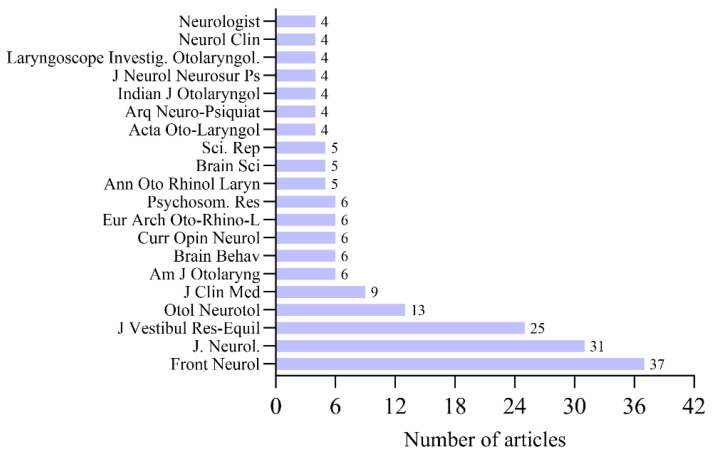
Top 20 most productive journals publishing PPPD research. Purple columns indicate the number of publications per journal (*Y*-axis: publication count). The distribution highlights the primary dissemination venues for PPPD evidence across neurology and neuro-otology outlets, providing a practical guide to where clinicians and researchers most commonly find diagnostic updates, comorbidity studies, and rehabilitation-oriented intervention evidence.

In terms of dissemination channels, Frontiers in Neurology leads the field with 37 publications, followed closely by the Journal of Neurology (31 papers) and the Journal of Vestibular Research-Equilibrium & Orientation (25 papers). The most productive journals are detailed in Figure 2, providing researchers with a strategic map of the primary venues for discourse in this domain. This distribution also reflects where PPPD evidence is most frequently consolidated and updated, which is particularly relevant for clinicians seeking high-yield sources for diagnostic developments, vestibular migraine overlap, vestibular rehabilitation strategies, and patient-reported outcome measures.

#### 3.1.2. The Intellectual Lineage and Vein of Research

**Figure 3:**
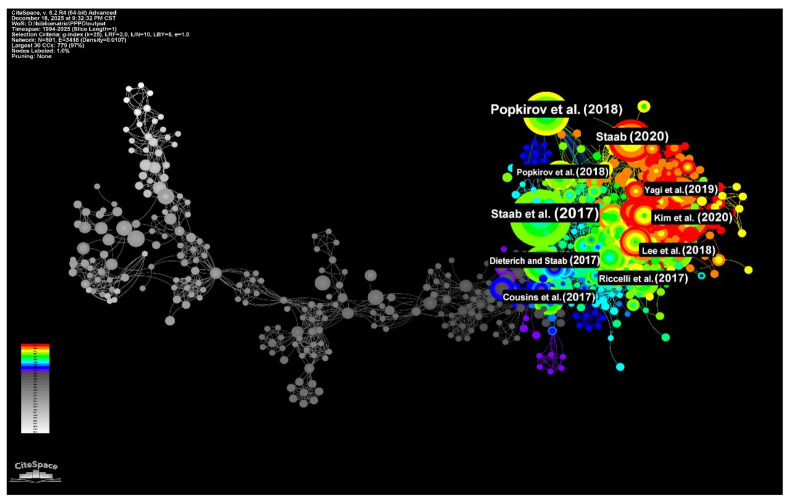
Document co-citation network and intellectual structure of PPPD research (1994–2025). The network comprises 801 nodes and 3418 links, visualizing the interconnectivity of influential publications. The color gradient from left (white/gray) to right (red) represents the temporal progression from 1994 to 2025. Nodes represent individual publications, where the size of each node reflects its co-citation frequency—a proxy for scientific impact. Links between nodes indicate that two papers are cited together in subsequent literature, forming the “intellectual lineage” of the field. Structurally, the map illustrates the organic evolution from early foundational theories (white/gray roots) toward specialized, post-consensus branches (red), highlighting the transition from descriptive phenomenology to standardized diagnostic framing and multimodal management. Key publications highlighted in the network include: Staab et al. (2017) [4], Popkirov et al. (2018) [25], and Staab (2020) [26], which command 109, 61, and 59 co-citations, respectively. These are flanked by significant works from Dieterich and Staab (2017) [3], Kim et al. (2020) [5], Riccelli et al. (2017) [27], Lee et al. (2018) [8], Yagi et al. (2019) [28], Cousins et al. (2017) [29], and Popkirov et al. (2018) [25].

To elucidate the structural evolution of the field over the past three decades, we constructed a document co-citation network (801 nodes, 3418 links) that visualizes the dense interconnectivity of the literature (Figure 3). The topology of this network strikingly resembles the organic growth of a tree. The “root system,” formed by literature from the early period (1994–2014) and marked in gray, exhibits high node density and rich connectivity, providing the foundational theories that nourish the field’s development. Moving into the intermediate phase (2015–2020), the network—marked in blue—begins to branch out, representing the diversification of research themes. In the most recent period (2021–2025), these branches have further differentiated into tight, specialized clusters, indicative of a field that is simultaneously consolidating consensus and exploring new sub-disciplines. In practical terms, this structure indicates a shift from early descriptive constructs toward post-consensus thematic diversification, particularly in comorbidity characterization, neurobiological models, rehabilitation protocols, and quality-of-life assessment.

Anchoring this network are several high-impact works that dominate in terms of co-citation frequency. The most prominent contributions include the study by Staab et al. (2017) [4], Popkirov et al. (2018) [25], and Staab (2020) [26], which command 109, 61, and 59 co-citations, respectively. These are flanked by significant works from Dieterich and Staab (2017) [3], Kim et al. (2020) [5], Riccelli et al. (2017) [27], Lee et al. (2018) [8], Yagi et al. (2019) [28], Cousins et al. (2017) [29], and Popkirov et al. (2018) [25]. This structural differentiation is further corroborated by the reference timeline analysis presented later in the study. These highly co-cited publications collectively represent the core evidence base that clinicians most frequently rely on for diagnostic criteria, differential diagnosis, mechanistic hypotheses, and multimodal management recommendations.

Complementing this network analysis, we employed HistCite Pro 2.1 to map the historiographic lineage of the research articles. Table 2 highlights the field’s milestone publications based on citation metrics, where node size correlates with bibliographic importance and linkage density reflects betweenness centrality. The three most impactful papers identified through this analysis are: Diagnostic criteria for PPPD: Consensus document of the committee for the Classification of Vestibular Disorders of the Bárány Society; Expanding the differential diagnosis of chronic dizziness; Functional dizziness: from phobic postural vertigo and chronic subjective dizziness to PPPD.

#### 3.1.3. Scientific Cooperation Networks

**Figure 4:**
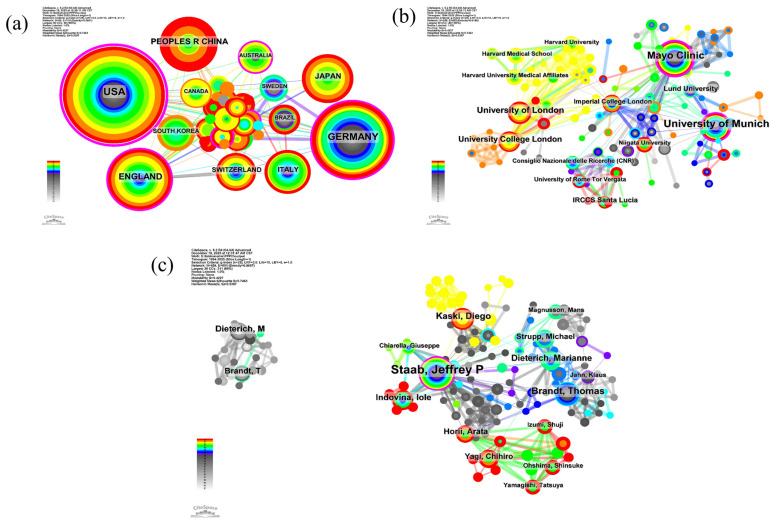
Scientific cooperation networks in PPPD research (1994–2025). (**a**) Country collaboration network; (**b**) institutional collaboration network; (**c**) author collaboration network. Node size represents the frequency of co-occurrence, and links represent collaborative relationships. The networks visualize the international and cross-institutional structure underpinning PPPD research, indicating where influential consensus-building and multicenter clinical studies are concentrated and where collaboration pathways may facilitate external validation of diagnostic criteria and treatment protocols.

The global landscape of research on PPPD is defined by robust collaboration across national, institutional, and individual dimensions, as evidenced by the extensive network of nodes and links in Figure 4.

At the national level (Figure 4a), the collaboration network (50 nodes, 114 links) is anchored by the United States, which exhibits the largest node size, followed by significant contributions from Germany, the United Kingdom, and China. Institutional cooperation (Figure 4b) is similarly extensive (258 nodes, 485 links), with the University of Munich, the Mayo Clinic, the University of London, and University College London emerging as the primary hubs of research activity. The author collaboration network (Figure 4c) highlights the dense interpersonal connections driving the field forward. Key opinion leaders such as Staab, Jeffrey P.; Brandt, Thomas; Kaski, Diego; and Dieterich, M., not only lead in publication volume but are also centrally positioned within the web of collaboration, underscoring the highly cooperative nature of the research community in this domain. Such network centrality often corresponds to the generation and dissemination of consensus-oriented clinical concepts and standardized assessment approaches, thereby influencing how PPPD is recognized and managed across centers.

### 3.2. Dynamic Evolution of Research Hotspots and Frontiers

#### 3.2.1. Temporal Shifts in Disciplinary Attention

**Figure 5:**
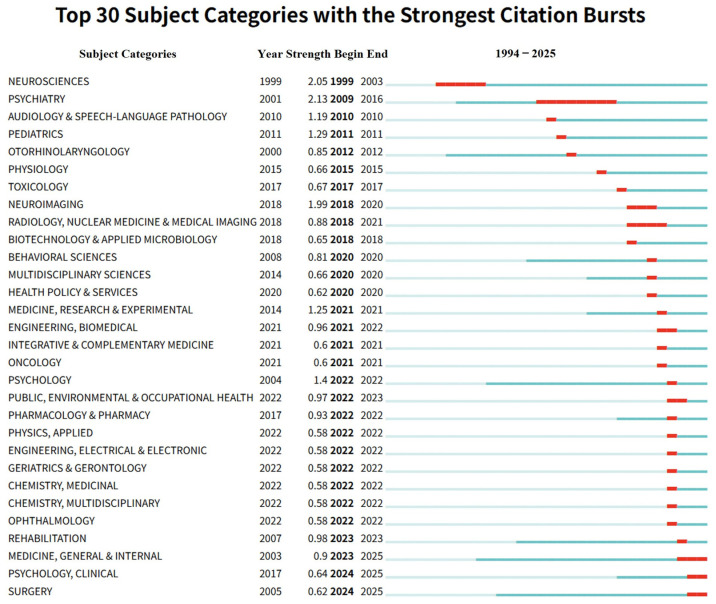
Top 30 subject categories with the strongest citation bursts (1994–2025). The blue timeline denotes the observation window, while red segments indicate burst periods of heightened attention for each category. “Year” marks the first occurrence, “Strength” reflects burst intensity, and “Begin/End” define the burst duration. The shifting burst profile illustrates the field’s movement from earlier psychiatry-centered attention toward a broader multidisciplinary clinical landscape involving neurosciences, otorhinolaryngology, rehabilitation-related categories, and general/internal medicine, paralleling the evolving understanding of PPPD as a functional neuro-otologic disorder with complex comorbidities.

The evolution of scientific interest across disciplines was analyzed using Kleinberg’s burst detection algorithm, identifying periods of intense activity. Between 1994 and 2025, citation bursts were detected in 36 of the 39 relevant subject categories, illustrating a dynamic shift in the intellectual center of gravity for the field. The temporal distribution of these bursts is visualized in Figure 5, where the blue line represents the observation period and the red segments denote the duration of the burst.

Historically, the category of Psychiatry exhibited the most profound influence, registering the highest burst strength (2.13) during the period from 2009 to 2016. This suggests that the early to mid-phase of research was heavily anchored in understanding the psychogenic dimensions of the disorder. However, as the field expanded after diagnostic standardization, the focus diversified significantly. Distinct bursts appeared in Neurosciences (1999–2003), Pediatrics (2011), and Physiology (2015), culminating in a recent surge in Medicine, General & Internal (2023–2025). This progression underscores the field’s transformation from a specialized niche into a multidisciplinary domain with broad clinical relevance. Notably, the current landscape (post-2024) is defined by active bursts in 20 distinct subject categories (Appendix A). The most prominent among these are Medicine, General & Internal (2023–2025), Surgery (2024–2025), and Sport Sciences (2024–2025). This recent shift implies an expanding recognition of PPPD across diverse medical and rehabilitative specialties. The category-level expansion supports the view that PPPD is increasingly being addressed as a cross-disciplinary clinical entity involving neurology, otorhinolaryngology, rehabilitation, and general medicine, rather than being confined to a single explanatory framework.

#### 3.2.2. Evolution of Key Terminology

**Figure 6:**
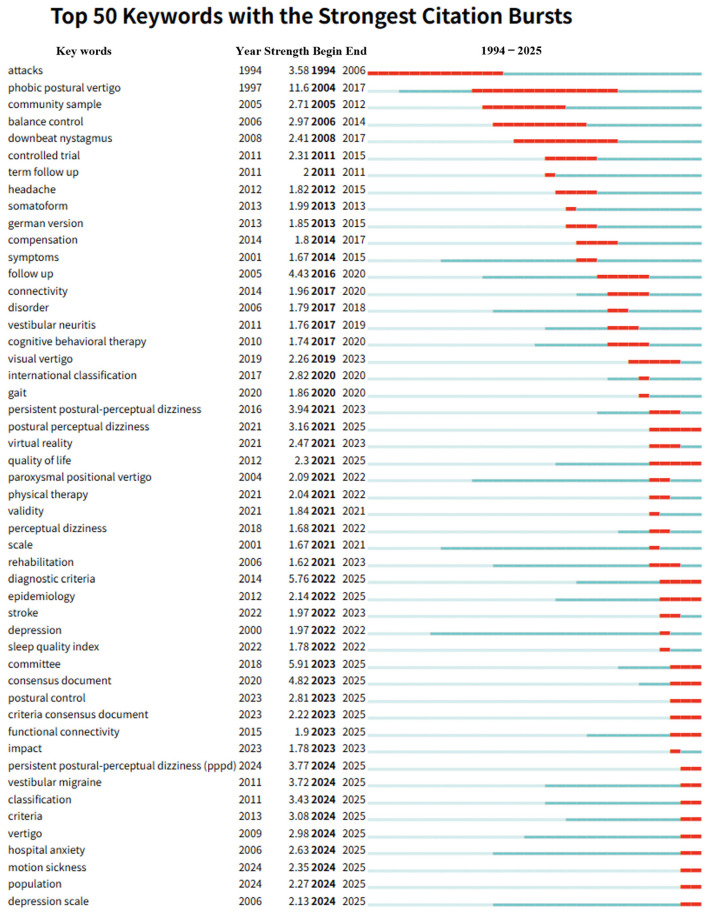
Top 50 keywords with the strongest citation bursts in PPPD research (1994–2025). The blue timeline denotes the observation window, while red segments indicate burst periods of heightened attention for each category. “Year” indicates the first appearance of the keyword; “Strength” reflects the intensity of the citation burst based on Kleinberg’s algorithm; “Begin” and “End” denote the duration of the burst period. Keywords are ranked by burst strength and displayed with their active time intervals, reflecting rapid increases in scholarly attention. The burst pattern demonstrates the conceptual evolution from historical phenomenological labels to standardized terminology and diagnostic framing, while also emphasizing contemporary fronts related to comorbidity, management strategies, and patient-centered outcome assessment.

At a more granular level, we analyzed the burst dynamics of 430 keywords to map the conceptual trajectory of the field from 1994 to 2025. Figure 6 highlights the top 50 keywords with the strongest citation bursts. The burst timeline provides an intuitive proxy for when specific diagnostic labels, comorbidity constructs, and management approaches gained prominence in the field.

The early terminology was dominated by specific phenomenological descriptions. “Phobic postural vertigo” exhibited the highest historical burst strength (11.6), dominating the literature from 2004 to 2017. This reflects the foundational era where the disorder was primarily conceptualized through the lens of phobic vertigo. In contrast, the lexicon of the modern era has shifted towards standardization and consensus. Recent high-strength bursts include “Committee” (strength 5.91; 2023–2025) and “Diagnostic criteria” (strength 5.76; 2022–2025), signaling a concerted global effort to formalize diagnosis.

Looking toward the future, we identified 20 keywords that remain in an active burst state as of 2025, serving as predictive indicators of emerging research frontiers (Appendix A). Key terms include “Diagnostic criteria” (strength 5.76), “Consensus document” (strength 4.82), “PPPD” (strength 3.77), and “VM” (strength 3.72). The sustained prominence of these terms suggests that refining diagnostic precision and exploring comorbidities, particularly VM, will constitute the core of the research agenda in the coming years. From a clinical perspective, this aligns with growing emphasis on structured diagnostic criteria, systematic evaluation of vestibular migraine overlap, and intervention studies integrating VRT with psychological and pharmacological strategies.

The disciplinary and conceptual frontiers currently shaping the field are further quantified in Table A1. This table highlights subject categories and keywords that maintain active citation bursts as of 2025, representing the most immediate research priorities.

#### 3.2.3. Landmark Literature and Intellectual Pivots

A comprehensive analysis of citation kinetics identified 758 articles with significant citation bursts. Table 3 details the top 30 references that have shaped the discourse between 1994 and 2025. Among these, three seminal works warrant detailed examination due to their profound impact on the field’s conceptual framework.

##### Standardization of Diagnosis

The publication with the highest citation burst, persisting from 2018 to 2022, is the consensus document titled “Diagnostic criteria for PPPD: Consensus document of the committee for the Classification of Vestibular Disorders of the Bárány Society” [4]. This landmark paper formally integrated PPPD into the International Classification of Vestibular Disorders (ICVD). It synthesized three decades of research on precursor concepts—including phobic postural vertigo, space motion discomfort, visual vertigo, and chronic subjective dizziness—to define PPPD as a distinct clinical entity.

The consensus criteria characterize PPPD as a chronic functional vestibular disorder manifested by unsteadiness, dizziness, or non-spinning vertigo lasting three months or more. Symptoms are typically exacerbated by upright posture, active or passive motion, and exposure to complex visual stimuli. The etiology is described as a maladaptive neuro-otologic response, potentially triggered by peripheral or central vestibular events, medical conditions, or psychological distress. Pathophysiologically, recent evidence suggests functional alterations in postural control mechanisms and cortical integration failures in spatial orientation and threat assessment. Crucially, this document reclassified the condition as a functional disorder, distinct from structural or purely psychiatric pathologies.

##### The Functional Paradigm

The second highly influential work, “Functional dizziness: from phobic postural vertigo and chronic subjective dizziness to PPPD” [3], exhibited a boost in attention from 2018 to 2022. This review addresses the transition in nomenclature toward “functional dizziness,” a term replacing older labels like somatoform or psychogenic dizziness. It highlights that functional dizziness accounts for up to 10% of cases in neuro-otology centers. The study emphasizes the high prevalence of psychiatric comorbidities (nearly 50%) in patients with structural vestibular syndromes, particularly VM and Ménière’s disease.

The authors propose a mechanism involving a “postural threat response”, where a precipitating event triggers anxiety-mediated changes in postural strategy, such as co-contraction of leg muscles and hyper-vigilance to body motion. Personality traits like high neuroticism are identified as risk factors. The paper argues that early diagnosis is critical to preventing chronicity and advocates for a multimodal treatment approach combining patient education, VRT, cognitive–behavioral therapy (CBT), and pharmacotherapy.

##### Clinical Management and Recognition

The third pivotal article, “ PPPD: a common, characteristic and treatable cause of chronic dizziness” [25], maintained a citation burst from 2019 to 2023. Upon publication, this work played a crucial role in clinical dissemination. It describes PPPD as a recognizable syndrome resulting from a long-term maladaptive adaptation of the brain and vestibular system to a triggering event. The authors of that study clarify that while standard diagnostic tests and neuroimaging typically yield negative results, the diagnosis is positive based on characteristic history and symptoms. Secondary complications often include functional gait disorders, anxiety, and avoidance behaviors. The paper reinforces the treatability of the condition through targeted physical therapy, serotonergic medication, and CBT, offering a hopeful prognosis for a condition previously considered refractory.

##### Emerging High-Impact Works

The analysis of the most recent literature (post-2025) reveals 90 papers currently experiencing citation bursts. The top 20, ranked by burst strength, are presented in Table 4. This cohort includes 4 review articles and 16 original research articles. The immediate impact of these publications suggests they are addressing critical knowledge gaps. The reviews provide essential theoretical guidance, while the original articles offer practical applications and empirical data. Rather than implying biological maturity, this pattern suggests a field that is conceptually consolidated and rapidly expanding, where high-level synthesis and targeted empirical studies are developing in parallel to refine diagnostic practice and optimize multimodal management. This bifurcation indicates a mature field that values both high-level synthesis and rigorous experimental validation, guiding researchers toward the most impactful avenues for future investigation.

### 3.3. Emerging Trends and Research Frontiers

#### 3.3.1. The Temporal Evolution of Conceptual Clusters

**Figure 7:**
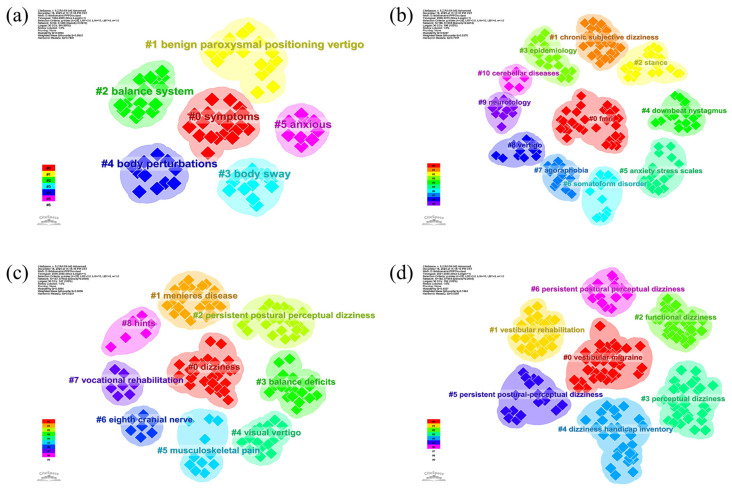
Keyword cluster snapshots across four developmental phases of PPPD research: (**a**) 1994–2005; (**b**) 2006–2015; (**c**) 2016–2020; (**d**) 2021–2025. Each snapshot shows the structure of co-occurring research themes within the corresponding period. The progression from early symptom-focused and differential-diagnosis clusters toward clusters centered on comorbidity characterization, rehabilitation interventions, and quality-of-life measurement reflects a transition from descriptive framing to clinically actionable research priorities, including standardized diagnosis, multimodal management, and outcome-based evaluation.

Keywords serve as the semantic DNA of scientific literature; their interrelationships and clustering behavior reveal the underlying intellectual structure of a field. By analyzing these semantic clusters over time, we can trace the distinct sub-domains that have shaped the study of PPPD. We stratified the past three decades into four distinct developmental phases, generating a series of cluster snapshots visualized in Figure 7.

The foundational phase (1994–2005, Figure 7a) was characterized by a limited dataset of 23 publications, which coalesced into six primary clusters, including symptoms (#0), benign paroxysmal positioning vertigo (#1), and balance system (#2). This era was largely defined by the exploration of basic symptomatology and differential diagnosis. The intermediate phase (2006–2015, Figure 7b) witnessed an expansion to 63 publications and a diversification into 11 clusters. Key themes such as fMRI (#0) and chronic subjective dizziness (#1) emerged, signaling a pivotal shift toward exploring neuroimaging mechanisms and the precursor concepts of functional dizziness. The pre-consensus phase (2016–2020, Figure 7c) consolidated 75 publications into 9 clusters, where terminology began to stabilize around PPPD (#2) while retaining a focus on key comorbidities like Ménière’s disease (#1). The contemporary phase (2021–2025, Figure 7d) represents a significant leap in research activity, comprising 209 publications that formed 7 distinct clusters. While foundational terms like PPPD remain central, the research focus has shifted markedly toward management and comorbidity. New high-density clusters have emerged, most notably VM (#0), VRT (#1), and DHI (#4). This evolution indicates that the field has moved beyond mere definition and is now deeply engaged in refining therapeutic protocols and understanding complex comorbidities. An in-depth examination of these contemporary clusters (Appendix A) highlights current priorities: Cluster #0 (VM, 35 articles) explores the intersection of vestibular and migrainous pathology; Cluster #1 (VRT, 33 articles) focuses on optimizing physical therapy protocols; and Cluster #4 (DHI, 28 articles) emphasizes the quantification of patient quality of life.

The structural characteristics of the clusters identified in the most recent research stage (2021–2025) are detailed in Table A2. High silhouette scores across these clusters confirm the robustness of the thematic division.

#### 3.3.2. Dynamics of Keyword Flow and Module Evolution

**Figure 8:**
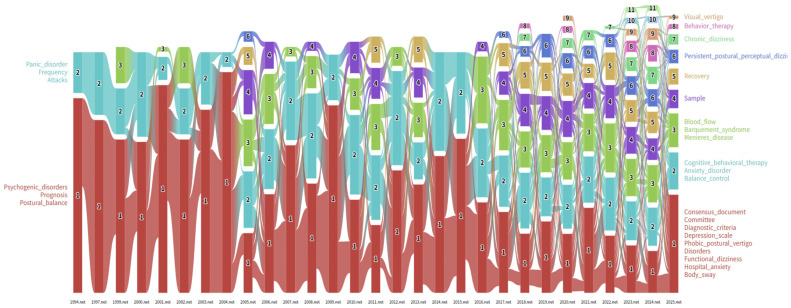
Alluvial map of keyword module evolution in PPPD research, 1994–2025. The *X*-axis represents time slices, and the *Y*-axis represents the count of modules; numbers denote the order of modules within each time slice, sorted by the number of nodes. Stream flows indicate how keywords split, merge, and persist across periods, thereby visualizing the continuity and transformation of major scientific themes. The persistence of modules associated with consensus-based terminology, comorbidity mechanisms, and rehabilitation-oriented interventions illustrates how the field consolidated diagnostic language while expanding clinically relevant lines of inquiry into treatment optimization and functional outcomes.

To visualize the life cycle of research topics, we employed an alluvial flow diagram (Figure 8), which maps how keywords split, merge, and evolve into distinct research modules over time. This “stream of science” visualization reveals that while some concepts have faded into obscurity, others have shown remarkable resilience or have metamorphosed into dominant research tributaries.

**Figure 9:**
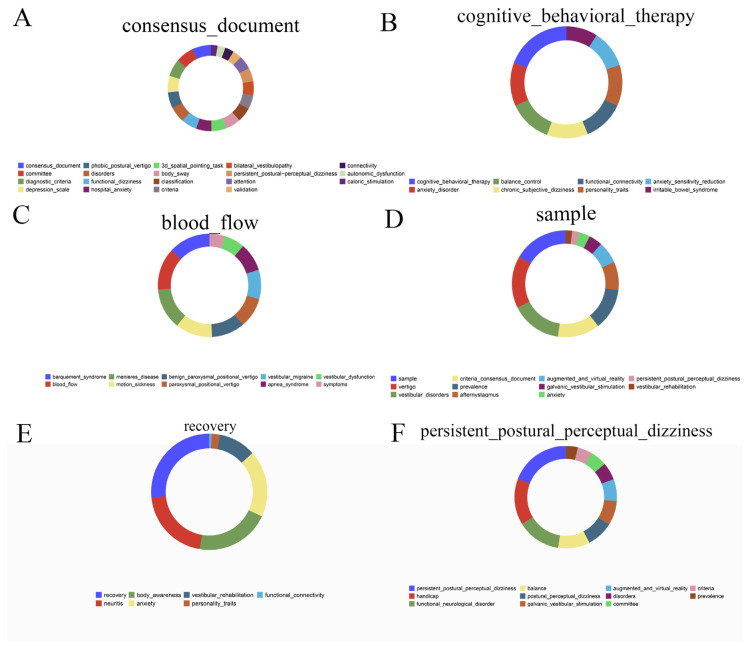
Core keywords within the dominant research modules in 2025. (**A**) Module 1; (**B**) Module 2; (**C**) Module 3; (**D**) Module 4; (**E**) Module 5; (**F**) Module 6. Each panel displays the most representative keywords composing a module, summarizing the dominant thematic structure at the current stage of the field. The module composition highlights the centrality of consensus-driven diagnostic framing, the sustained relevance of psychological and behavioral dimensions, and the growing prominence of rehabilitation and functional outcome constructs, consistent with a research agenda focused on diagnostic refinement, comorbidity-informed stratification, and multimodal intervention.

The analysis of the 2025 landscape identifies several robust modules (Appendix A). Module 1, the largest and most persistent tributary (marked in red), has been designated “Consensus Document.” Comprising 19 keywords such as committee and diagnostic_criteria (Figure 9A), this module underscores the field’s sustained focus on standardization and formal classification. Module 2, labeled “CBT,” aggregates eight keywords, including anxiety_disorder, balance_control, and personality_traits (Figure 9B), reflecting the established consensus on the psychosomatic and behavioral dimensions of treatment. Meanwhile, Module 3 (“Blood Flow”) encompasses ten keywords such as mal de debarquement syndrome and motion sickness (Figure 9C), suggesting ongoing investigations into vascular and motion-sensitivity mechanisms.

Further defining the current landscape, Module 4 (“Sample”) features 11 keywords, including vertigo and criteria_consensus_document (Figure 9D), likely representing epidemiological and large-cohort studies. Module 5 (“Recovery”) focuses on neuritis and body_awareness (Figure 9E), highlighting the recovery trajectory and proprioceptive aspects. Finally, Module 6 (“Persistent Postural-Perceptual Dizziness”) centers on handicap, functional_neurological_disorder, and balance (Figure 9F), cementing the core clinical identity of the disorder. Collectively, these modules represent the crystallized output of three decades of inquiry and are predictive of the research trajectory for the coming years.

To provide a chronological perspective on how core concepts have dominated the research landscape, Table A3 summarizes the most trafficked keywords within the top five modules annually from 1994 to 2025.

#### 3.3.3. Timeline Analysis and Landmark Literature

**Figure 10:**
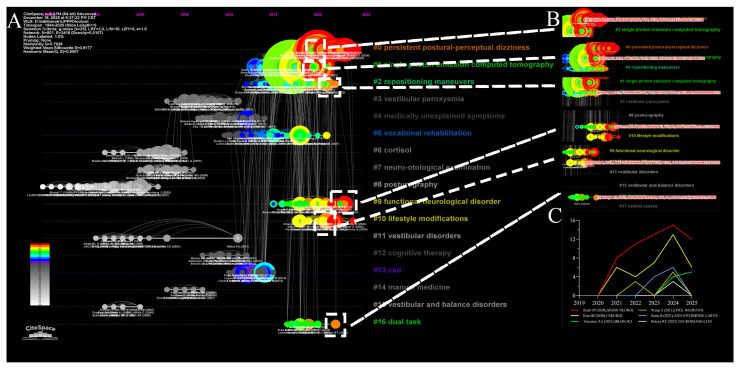
Reference cluster mapping and citation dynamics in PPPD research. (**A**) Timeline visualization of reference clusters, showing the emergence, persistence, and decline of thematic clusters over time; (**B**) burst references within selected clusters (#0, #1, #3, #9, #10, and #16), highlighting landmark publications that attracted rapidly increasing citations; (**C**) annual citation frequency distribution of burst references (*X*-axis: year; *Y*-axis: cited frequency). Together, these panels delineate the intellectual structure of PPPD research and identify pivotal works that shaped diagnostic consensus, clarified differential diagnosis and comorbidity patterns, and advanced management paradigms that integrate rehabilitation, psychological intervention, and patient-reported outcome measures.

The timeline visualization of reference clusters (Figure 10A) provides a temporal stratification of research topics, categorizing them into classic foundations, fading interests, and emerging frontiers. Classic clusters such as medically unexplained symptoms (#4), vocational rehabilitation (#5), and chronic subjective dizziness (#13) form the historical bedrock of the field. While no longer the primary focus, they remain structurally linked to modern concepts. Conversely, topics such as vestibular paroxysmia (#3), cortisol (#6), and manual medicine (#14) appear relatively obsolete, showing limited connectivity and ceased activity on the timeline. Most importantly, the analysis identifies “emerging frontiers”—topics that have remained active from their inception to the present. These include PPPD (#0), single photon emission computed tomography (#1), repositioning maneuvers (#2), functional neurological disorder (#9), lifestyle modifications (#10), and dual task (#16). The sustained activity in these clusters forecasts their role as the primary engines of future discovery.

The timeline also highlights specific “landmark nodes”—papers with high citation bursts that have fundamentally altered the field’s direction (Figure 10B). A cornerstone reference is the work by Staab (2020) [26] within Cluster #2, which solidified the status of PPPD following its 2017 inclusion in the International Classification of Vestibular Disorders. This work defined PPPD as a chronic functional vestibular disorder characterized by fluctuating unsteadiness and non-spinning vertigo, exacerbated by upright posture, motion, and visual stimuli. Crucially, it established a widely cited multimodal management framework—combining specialized VRT, serotonergic pharmacotherapy, and CBT—that has become a central reference point for contemporary treatment discussions.

Providing critical epidemiological context, Kim et al. (Cluster #1) conducted a massive retrospective analysis of 21,166 patients in a Korean dizziness clinic. The study identified PPPD as the second most common cause of dizziness (20.8%) after BPPV. Notably, PPPD was identified as the leading cause of dizziness in adults aged 19–64 (26.3%), highlighting its massive socioeconomic impact on the working-age population. Addressing the pediatric demographic, the study titled “PPPD in Children and Adolescents” (Cluster #2) filled a critical gap by characterizing the disorder in younger populations. It revealed that PPPD in children is frequently comorbid with BPPV and VM, often leading to significant school absenteeism, yet offers a hopeful prognosis with combined physical and behavioral therapies.

The complex relationship between PPPD and migraine is addressed by two key papers. The article “What’s in a Name? Chronic VM or Persistent Postural Perceptual Dizziness?” (Cluster #9) debates the classification of chronic dizziness, proposing that when VM transitions to a continuous state, it may be best conceptualized within the PPPD framework. Complementing this, the study “Migraine Features in Patients with PPPD” (Cluster #10) found that while over half of PPPD patients met the full criteria for migraine, many others exhibited partial features, suggesting that PPPD may exist on a spectrum with otologic migraine. Finally, exploring neuroanatomical correlates, the study “A link between frontal white matter integrity and dizziness in cerebral small vessel disease” (Cluster #16) demonstrated that elderly patients with idiopathic dizziness exhibit reduced fractional anisotropy in specific white matter tracts. This suggests that some cases of chronic dizziness may stem from disconnection in white matter networks responsible for balance and executive function, rather than peripheral vestibular failure. The citation distribution analysis (Figure 10C) indicates that these papers continue to accumulate citations rapidly, suggesting their influence will persist and likely shape the research narrative for years to come.

## 4. Discussion

The bibliometric topography mapped in this study reveals a striking “J-curve” trajectory in global research output on PPPD, culminating in a historic peak in 2024. This exponential growth reflects more than a mere accumulation of data; from a bibliometric perspective, it represents an intense shift in scholarly interest in neuro-otology—a transition from viewing chronic dizziness as a diagnosis of exclusion to recognizing it as a distinct, structurally defined functional neurological disorder [44]. Our analysis indicates that while the field germinated slowly during the era of “phobic postural vertigo”, the publication of the Bárány Society’s consensus criteria in 2017 served as a critical inflection point. This mirrors the “crystallization phase” often seen in medical taxonomy, where semantic unification catalyzes scientific productivity [26]. The sustained surge in citations for the 2017 consensus document confirms its role as the “Rosetta Stone” of the field, enabling cross-center validation and large-scale epidemiological studies that were previously impossible due to nomenclatural fragmentation [62].

Furthermore, the diversification of publication venues—expanding from specialized otolaryngology journals to broad-spectrum neurology and general medicine titles—suggests that PPPD is graduating from a niche subspecialty interest to a mainstream medical concern. This is clinically imperative, as recent epidemiological data suggest PPPD accounts for up to 20% of diagnoses in tertiary dizziness clinics, a burden comparable to that of migraine [52]. The bibliometric burst in “General Medicine” categories observed in our study (2023–2025) likely reflects an improved recognition pipeline in primary care, where practitioners are increasingly identifying the “three core symptoms” (unsteadiness, exacerbation by upright posture, and visual stimuli) rather than dismissing patients as purely anxious [63].

One of the most profound findings of our co-occurrence analysis is the migration of research focus from Psychiatry (dominant 2009–2016) to Neuroscience and Multidisciplinary Medicine (dominant 2020–2025). This bibliometric drift aligns perfectly with the evolving understanding of PPPD pathophysiology. Historically, patients were stigmatized under the “psychogenic” label, implying an imaginary or purely emotional etiology [51]. However, contemporary literature supports a “software, not hardware” model—a functional readaptation failure of the postural control system [64].

The thematic clusters identified in our analysis reflect a research trend that explores a ‘top-down’ mechanism where maladaptive predictive coding is hypothesized to lead to a stiffened postural strategy.”

(Original): “The bibliometric overlap suggests that the relationship between PPPD and VM is not merely coincidental but potentially syndromic or sequential [65]. Functional MRI (fMRI) studies published in the last three years have elucidated this mechanism, showing that PPPD patients exhibit decreased connectivity in the vestibular cortex but increased activity in the visual cortex during motion processing [66]. This “visual dependence”—a key term identified in our keyword analysis—explains the hypersensitivity to complex visual environments (visual vertigo) [67]. The shift in our data away from pure psychiatric keywords suggests the academic community now views anxiety not as the cause, but as a perpetuating factor and comorbidity that locks the brain into a high-alert state. This distinction is crucial for destigmatization and is driving the surge in neurobiological rather than purely psychodynamic research.

Our burst detection analysis identified “VM” as one of the strongest emerging frontiers (2021–2025). This highlights the most challenging differential diagnosis in current practice. The bibliometric overlap suggests that within current scientific discourse, the relationship between PPPD and VM is conceptualized as potentially syndromic or sequential. Recent evidence indicates that VM may act as a potent “precipitating event” for PPPD; the fluctuating vestibular errors caused by migraine attacks prevent the central compensation required to reset the internal model of stability.

Researchers are now exploring the shared genetic and neurochemical substrates of these two conditions. The “sensory disintegration” theory proposes that both VM and PPPD share a central hypersensitivity to sensory inputs (light, sound, motion), mediated by alterations in calcitonin gene-related peptide and serotonergic pathways [68]. The strong citation linkage between these clusters in our study supports the notion of a “vestibular–migraine–anxiety” triad. Clinically, this bibliometric trend validates the increasing use of migraine prophylactics (e.g., venlafaxine, nortriptyline) in PPPD management, even in the absence of headache, aiming to lower the sensory threshold [69]. Future research, as predicted by our “keyword burst” analysis, will likely focus on identifying clinically relevant biomarkers that can distinguish “pure” PPPD from “migrainous” PPPD to better inform stratified management approaches [70].

The evolution of keyword clusters from “symptom description” to “VRT” and “CBT” reflects the maturation of therapeutic protocols. Our analysis shows that “VRT” is currently a high-density node, distinct from general physical therapy. This aligns with recent randomized controlled trials demonstrating that generic balance exercises are insufficient for PPPD; instead, habituation exercises specifically targeting visual desensitization (optokinetic stimulation) are required [58]. Moreover, the emergence of keywords related to “Digital therapeutics” and “Virtual Reality” in the post-2023 dataset signals a new frontier. Traditional VRT often suffers from low adherence due to the monotony of exercises. Recent studies cited in our network suggest that VR-based interventions can simulate complex visual environments (e.g., supermarkets, busy streets) in a controlled setting, which is reported in current studies as a potential method to facilitate the ‘re-weighting’ of sensory inputs [71]. Additionally, the bursting of “CBT” highlights the consensus that dismantling the “fear of falling” loop is essential. The latest internet-delivered CBT (iCBT) trials have shown promise in making this specialized psychological support accessible, addressing the global shortage of psychotherapists trained in vestibular disorders [72]. The integration of VRT and CBT—often termed “hybrid therapy”—represents an increasingly prominent research theme and a highly prioritized therapeutic strategy within the recent literature [73].

The geographic analysis reveals a bipolar dominance of the United States (Mayo Clinic) and Germany (University of Munich), which have historically driven the theoretical framework of the field. While this concentration of expertise has been beneficial for establishing consensus, it introduces a potential bias. The clinical presentation of PPPD may vary across cultures due to differences in somatization patterns and the stigmatization of mental health symptoms [74]. However, our data shows a rising contribution from China and East Asia in the 2020–2025 period. This globalization is vital. Recent Asian cohorts have provided unique insights into the “visual-dominant” phenotype of PPPD, potentially linked to high-density urban living environments [40]. Promoting multi-center international collaborations, similar to those seen in the Bárány Society, will be essential to test the cross-cultural validity of the DHI and other patient-reported outcome measures highlighted in our keyword analysis [60].

### Study Limitations

Despite the progress, our timeline analysis identifies “gaps” in the current map—specifically, the lack of molecular biomarkers. While “fMRI” and “posturography” appear as clusters, keywords related to genetics or blood-based biomarkers are notably absent from the high-burst list. This represents the next logical step for the field [75]. Investigating the genetic polymorphisms related to serotonin transport or stress response axes could help explain why only a subset of patients develop PPPD after a vestibular insult [76]. Furthermore, the integration of Artificial Intelligence into posturography analysis—barely visible in current keywords but likely to burst soon—could offer objective diagnostic metrics to complement subjective history-taking [77]. This study is subject to inherent bibliometric limitations. First, the data collection was restricted to the WoS. While WoS is the gold standard for the specific citation-based algorithms used in CiteSpace and HistCite, it does not cover the entirety of the medical literature. Consequently, specific clinical studies, case reports, or regional guidelines indexed exclusively in PubMed/Medline or Scopus may have been omitted. This exclusion means that while the “intellectual structure” mapped here is robust, certain clinical nuances or niche ENT perspectives might be underrepresented. Future studies should consider a multi-database approach to capture a broader spectrum of clinical ENT and neurology literature. Moreover, the analysis may be influenced by potential indexing bias between “Author Keywords” (reflecting the researchers’ specific focus) and “Keywords Plus” (algorithmically generated by WoS based on cited references). Discrepancies between these two sources can occasionally prioritize broader diagnostic labels over more specialized clinical terminology. Second, citation-based metrics such as citation counts and betweenness centrality can be affected by regional publication clusters or potential self-citation effects within specific research groups, which may slightly skew the perceived global impact of certain countries or institutions. Additionally, the citation burst analysis has a lag time; very recent groundbreaking papers from late 2024 or 2025 may not yet have accumulated sufficient citations to appear as major nodes, despite their potential importance. Finally, bibliometric analysis provides a quantitative map of scientific discourse rather than a qualitative evaluation of the scientific rigor of individual studies. It is essential to emphasize that while bibliometric metrics identify thematic prominence and collaborative structures, they reflect publication attention and research intensity rather than providing direct evidence of clinical efficacy or pathophysiological validation. Bibliometrics can map where the scientific community is looking, but it cannot independently validate underlying biological mechanisms or confirm the superiority of specific therapeutic protocols. Therefore, the results reflect the “popularity” and “impact” of research topics within the academic community, which should be interpreted alongside clinical expertise, traditional systematic reviews, randomized controlled trials, and professional clinical guidelines.

## 5. Conclusions

The intellectual lineage of PPPD research has transitioned from a fragmented collection of phenomenological descriptions toward a unified, functional neurological framework. Our bibliometric mapping underscores that the 2017 Bárány Society consensus served as a critical catalyst, shifting the scholarly center of gravity from psychiatry toward multidisciplinary neuroscience and otorhinolaryngology. Current research frontiers are characterized by an intense focus on diagnostic refinement, comorbidity exploration (particularly vestibular migraine), and the optimization of multimodal rehabilitation strategies. While the field has achieved conceptual stabilization, the bibliometric evidence points toward an emerging emphasis on identifying objective biomarkers to further consolidate the pathophysiological understanding of this disorder.

## Figures and Tables

**Table 1:** Quantitative overview of literature on Persistent Postural-Perceptual Dizziness (1994–2025).

Categories	Publication	Articles	Review	Authors	Institutions	Journals	Subject Categories
Amount	370	304	66	1290	537	146	39

**Table 2:** Bibliometric details of the top 30 landmark publications ranked by Local Citation Score (LCS).

NO.	Article Information	Journal	LCS	GCS
92	Diagnostic criteria for persistent postural-perceptual dizziness (PPPD): Consensus document of the committee for the Classification of Vestibular Disorders of the Barany Society	J Vestibul Res-Equil	208	477
29	Expanding the differential diagnosis of chronic dizziness	Arch Otolaryngol	81	154
97	Functional dizziness: from phobic postural vertigo and chronic subjective dizziness to persistent postural-perceptual dizziness	Curr Opin Neurol	77	128
141	Persistent Postural-Perceptual Dizziness	Semin Neurol	60	89
56	Cognitive behavior therapy for chronic subjective dizziness: a randomized, controlled trial	Am J Otolaryng	57	81
70	Anxious, introverted personality traits in patients with chronic subjective dizziness	J Psychosom Res	50	94
80	Clinical characteristics of patients with persistent postural-perceptual dizziness	Braz J Otorhinolar	49	67
26	Treatment of phobic postural vertigo—A controlled study of cognitive-behavioral therapy and self-controlled desensitization	J Neurol	46	76
110	Altered brain function in persistent postural perceptual dizziness: A study on resting state functional connectivity	Hum Brain Mapp	45	75
4	Patients with somatoform phobic postural vertigo: the more difficult the balance task, the better the balance performance	Neurosci Lett	42	68
78	Retrospective review and telephone follow-up to evaluate a physical therapy protocol for treating persistent postural-perceptual dizziness: A pilot study	J Vestibul Res-Equil	42	58
20	Chronic dizziness and anxiety—Effect of course of illness on treatment outcome	Arch Otolaryngol	41	79
18	Phobic postural vertigo—A long-term follow-up (5 to 15 years) of 106 patients	J Neurol	40	73
64	Inadequate interaction between open- and closed-loop postural control in phobic postural vertigo	J Neurol	39	67
91	Analysis of the characteristics of persistent postural-perceptual dizziness: A clinical-based study in China	Int J Audiol	39	45
87	Posturographic profile of patients with persistent postural-perceptual dizziness on the sensory organization test	J Vestibul Res-Equil	38	51
61	Cognitive behavior therapy for chronic subjective dizziness: longer-term gains and predictors of disability	Am J Otolaryng	37	58
30	One-year follow-up of cognitive behavioral therapy for phobic postural vertigo	J Neurol	37	51
124	Vestibular Rehabilitation Therapy Outcomes in Patients with Persistent Postural-Perceptual Dizziness	Ann Oto Rhinol Laryn	37	52
150	Etiologic distribution of dizziness and vertigo in a referral-based dizziness clinic in South Korea	J Neurol	36	114
22	What accounts for vertigo one year after neuritis vestibularis—anxiety or a dysfunctional vestibular organ?	J Psychiatr Res	36	85
3	Increased body sway at 3.5–8 Hz in patients with phobic postural vertigo	Neurosci Lett	34	83
73	Gait characteristics of patients with phobic postural vertigo: effects of fear of falling, attention, and visual input	J Neurol	34	63
1	PHOBIC POSTURAL VERTIGO—A FIRST FOLLOW-UP	J Neurol	33	85
99	Cerebral gray matter changes in persistent postural perceptual dizziness	J Psychosom Res	31	50
32	Chronic subjective dizziness	Acta Oto-Laryngol	29	48
88	Chronic subjective dizziness: Analysis of underlying personality factors	J Vestibul Res-Equil	29	44
2	Course of illness in phobic postural vertigo	Acta Neurol Scand	29	36
103	Brain responses to virtual reality visual motion stimulation are affected by neurotic personality traits in patients with persistent postural-perceptual dizziness	J Vestibul Res-Equil	29	42
159	Primary or secondary chronic functional dizziness: does it make a difference? A DizzyReg study in 356 patients	J Neurol	28	45

LCS: Local Citation Score (citations within this dataset); GCS: Global Citation Score (total citations in Web of Science).

**Table 3:** References with significant citation bursts across different periods. “Year” indicates publication year; “Strength” measures the burst intensity; “Begin” and “End” mark the period of peak scholarly impact. Blue indicates the literature search time range, while red represents the period during which the literature emerged.

References	Year	Strength	Begin	End	1994–2025
Querner V, 2000, NEUROSCI LETT, V285, P21, DOI 10.1016/S0304-3940(00)01008-9, DOI [30]	2000	5.79	2001	2005	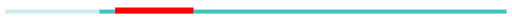
Staab JP, 2003, LARYNGOSCOPE, V113, P1714, DOI 10.1097/00005537-200310000-00010, DOI [31]	2003	5.87	2005	2006	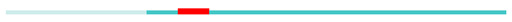
Staab Jeffrey P, 2012, CONTINUUM (MINNEAP MINN), V18, P1118, DOI 10.1212/01.CON.0000421622.56525.58, DOI [32]	2012	11.11	2014	2017	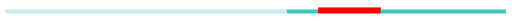
Staab JP, 2014, J PSYCHOSOM RES, V76, P80, DOI 10.1016/j.jpsychores.2013.11.008, DOI [33]	2014	8.5	2014	2019	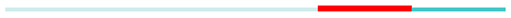
Wuehr M, 2013, J NEUROL, V260, P1314, DOI 10.1007/s00415-012-6797-7, DOI [34]	2013	7.12	2015	2018	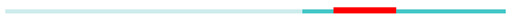
Mahoney AEJ, 2013, AM J OTOLARYNG, V34, P115, DOI 10.1016/j.amjoto.2012.09.013, DOI [35]	2013	6.52	2015	2018	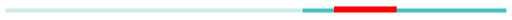
Indovina I, 2015, FRONT BEHAV NEUROSCI, V9, 334, DOI 10.3389/fnbeh.2015.00334, DOI [36]	2015	8.52	2017	2020	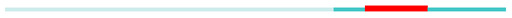
Cousins S, 2017, ANN CLIN TRANSL NEUR, V4, P340, DOI 10.1002/acn3.386, DOI [29]	2017	8.11	2017	2022	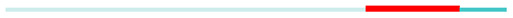
Holle D, 2015, PLOS ONE, V10, e0142468, DOI 10.1371/journal.pone.0142468, DOI [37]	2015	6	2017	2020	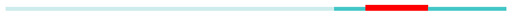
Söhsten E, 2016, J VESTIBUL RES-EQUIL, V26, P319, DOI 10.3233/VES-160583, DOI [38]	2016	5.69	2017	2021	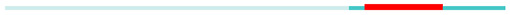
Van Ombergen A, 2017, NEUROIMAGE-CLIN, V14, P538, DOI 10.1016/j.nicl.2017.02.020, DOI [39]	2017	5.57	2017	2022	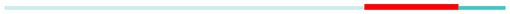
Staab JP, 2017, J VESTIBUL RES-EQUIL, V27, P191, DOI 10.3233/VES-170622, DOI [4]	2017	35.24	2018	2022	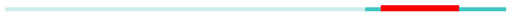
Dieterich M, 2017, CURR OPIN NEUROL, V30, P107, DOI 10.1097/WCO.0000000000000417, DOI [3]	2017	14.75	2018	2022	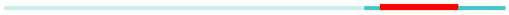
Riccelli R, 2017, FRONT NEUROL, V8, 529, DOI 10.3389/fneur.2017.00529, DOI [27]	2017	9.97	2018	2022	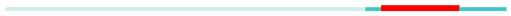
Yan ZH, 2017, INT J AUDIOL, V56, P33, DOI 10.1080/14992027.2016.1211763, DOI [40]	2017	7.76	2018	2022	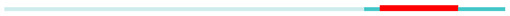
Wurthmann S, 2017, J PSYCHOSOM RES, V103, P95, DOI 10.1016/j.jpsychores.2017.10.007, DOI [41]	2017	6.51	2018	2022	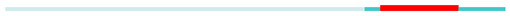
Chiarella G, 2016, J VESTIBUL RES-EQUIL, V26, P403, DOI 10.3233/VES-160590, DOI [42]	2016	5.68	2018	2021	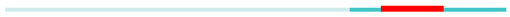
Popkirov Stoyan, 2018, PRACT NEUROL, V18, P5, DOI 10.1136/practneurol-2017-001809, DOI [25]	2018	12.71	2019	2023	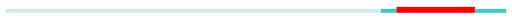
Lee JO, 2018, HUM BRAIN MAPP, V39, P3340, DOI 10.1002/hbm.24080, DOI [8]	2018	9.45	2019	2022	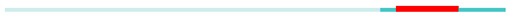
Wuehr M, 2017, NEUROLOGY, V88, P284, DOI 10.1212/WNL.0000000000003516, DOI [43]	2017	7.14	2019	2021	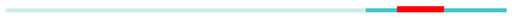
Popkirov S, 2018, CURR TREAT OPTION NE, V20, P0, DOI 10.1007/s11940-018-0535-0, DOI [44]	2018	8.32	2020	2023	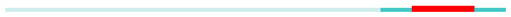
Staab JP, 2020, SEMIN NEUROL, V40, P130, DOI 10.1055/s-0039-3402736, DOI [26]	2020	6.78	2021	2025	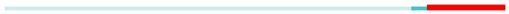
Powell G, 2020, NEUROLOGY, V94, PE1929, DOI 10.1212/WNL.0000000000009373, DOI [45]	2020	5.82	2021	2025	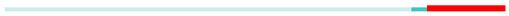
Passamonti L, 2018, J VESTIBUL RES-EQUIL, V28, P369, DOI 10.3233/VES-190653, DOI [46]	2018	5.57	2021	2023	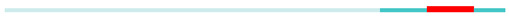
Yagi C, 2019, OTOL NEUROTOL, V40, PE747, DOI [28] 10.1097/MAO.0000000000002325, DOI	2019	7.67	2022	2025	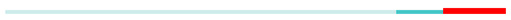
Herdman D, 2022, J NEUROL, V269, P4753, DOI 10.1007/s00415-022-11107-w, DOI [47]	2022	7.07	2023	2025	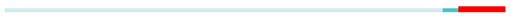
Kim HJ, 2020, J NEUROL, V267, P2252, DOI 10.1007/s00415-020-09831-2, DOI [5]	2020	5.6	2023	2025	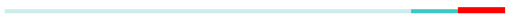
Staab JP, 2023, NEUROL CLIN, V41, P647, DOI 10.1016/j.ncl.2023.04.003, DOI [48]	2023	8.02	2024	2025	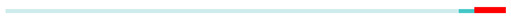
Trinidade A, 2023, J NEUROL NEUROSUR PS, V94, P904, DOI 10.1136/jnnp-2022-330196, DOI [49]	2023	6.82	2024	2025	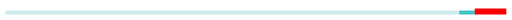
Indovina I, 2021, J CLIN MED, V10, P0, DOI 10.3390/jcm10184274, DOI [9]	2021	6.06	2024	2025	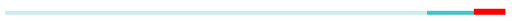

**Table 4:** Recent references with active citation bursts as of 2025. “Begin/End” indicates the burst duration; “Strength” reflects the magnitude of increase in citations; “Type” denotes the study design.

Begin	End	Strength	Year	Type	Title
2024	2025	8.02	2023	Review	Persistent Postural-Perceptual Dizziness: Review and Update on Key Mechanisms of the Most Common Functional Neuro-otologic Disorder [48]
2022	2025	7.67	2019	Article	A Validated Questionnaire to Assess the Severity of Persistent Postural-Perceptual Dizziness (PPPD): The Niigata PPPD Questionnaire (NPQ) [28]
2024	2025	6.82	2023	Review	Predictors of persistent postural-perceptual dizziness (PPPD) and similar forms of chronic dizziness precipitated by peripheral vestibular disorders: a systematic review [49]
2021	2025	6.78	2020	Review	Persistent Postural-Perceptual Dizziness [26]
2024	2025	6.06	2021	Review	Brain Correlates of Persistent Postural-Perceptual Dizziness: A Review of Neuroimaging Studies [9]
2021	2025	5.82	2020	Article	Persistent postural perceptual dizziness is on a spectrum in the general population [45]
2023	2025	5.6	2020	Article	Etiologic distribution of dizziness and vertigo in a referral-based dizziness clinic in South Korea [5]
2023	2025	5.56	2022	Article	Presence of exacerbating factors of persistent perceptual-postural dizziness in patients with vestibular symptoms at initial presentation [50]
2023	2025	5.37	2022	Article	Persistent Postural-Perceptual Dizziness (PPPD) from Brain Imaging to Behaviour and Perception [51]
2023	2025	5.28	2021	Article	Persistent Postural-Perceptual Dizziness: Precipitating Conditions, Co-morbidities and Treatment with Cognitive Behavioral Therapy [52]
2023	2025	5.03	2021	Article	Effect of vestibular exercise and optokinetic stimulation using virtual reality in persistent postural-perceptual dizziness [53]
2023	2025	4.63	2021	Article	Migraine Features in Patients with Persistent Postural-Perceptual Dizziness [54]
2024	2025	4.44	2022	Article	Comparison of Clinical Balance and Visual Dependence Tests in Patients with Chronic Dizziness with and Without Persistent Postural-Perceptual Dizziness: A Cross-Sectional Study [21]
2023	2025	4.36	2023	Article	Home-based Vestibular Rehabilitation: A Feasible and Effective Therapy for Persistent Postural Perceptual Dizziness (A Pilot Study) [55]
2022	2025	4.36	2021	Article	Assessment of Potential Risk Factors for the Development of Persistent Postural-Perceptual Dizziness: A Case-Control Pilot Study [56]
2023	2025	4.36	2023	Article	Non-pharmacological interventions for persistent postural-perceptual dizziness (PPPD) [57]
2021	2025	4.2	2019	Article	Vestibular Rehabilitation Therapy Outcomes in Patients with Persistent Postural-Perceptual Dizziness [58]
2023	2025	4.02	2023	Article	Postural misperception: a biomarker for persistent postural perceptual dizziness [59]
2024	2025	4	2023	Article	Persistent Postural-Perceptual Dizziness (PPPD) and quality of life: a cross-sectional study [60]
2024	2025	3.99	2022	Article	The impact of disease duration in persistent postural-perceptual dizziness (PPPD) on the quality of life, dizziness handicap and mental health [61]

## Data Availability

The original contributions presented in this study are included in the Supplementary Material. Further inquiries can be directed to the corresponding author.

## Supplementary Materials

The following supporting information can be downloaded at https://www.mdpi.com/article/10.3390/audiolres16020052/s1. The original downloaded plain text files. The cleaned and deduplicated dataset used for visualization. Working directories created during visualization, including related work records and software operation logs.

## Abbreviations

The following abbreviations are used in this manuscript:

PPPD	Persistent Postural-Perceptual Dizziness
VM	Vestibular Migraine
GCST	Vestibular Rehabilitation
CBT	Cognitive Behavioral Therapy
DHI	Dizziness Handicap Inventory
BPPV	Benign Paroxysmal Positional Vertigo
fMRI	Functional Magnetic Resonance Imaging
LCS	Local Citation Score
GCS	Global Citation Score

## Appendix A

**Table A1:** The top 10 subject categories and keywords with citation bursts ending in 2025.

Subject Category Bursts					Keywords Bursts				
Begin	End	Strength	Year	Entity	Begin	End	Strength	Year	Entity
2023	2025	0.9	2003	MEDICINE, GENERAL & INTERNAL	2022	2025	5.76	2014	diagnostic criteria
2024	2025	0.62	2005	SURGERY	2023	2025	4.82	2020	consensus document
2024	2025	0.38	2007	SPORT SCIENCES	2024	2025	3.77	2024	persistent postural-perceptual dizziness (pppd)
2024	2025	0.64	2017	PSYCHOLOGY, CLINICAL	2024	2025	3.72	2011	vestibular migraine
-	-	-	-	-	2024	2025	3.43	2011	classification
-	-	-	-	-	2021	2025	3.16	2021	postural perceptual dizziness
-	-	-	-	-	2024	2025	3.08	2013	criteria
-	-	-	-	-	2024	2025	2.98	2009	vertigo
-	-	-	-	-	2023	2025	2.81	2023	postural control
-	-	-	-	-	2024	2025	2.63	2006	hospital anxiety

**Table A2:** Summary of keyword clusters for the most recent stage (2021–2025).

ClusterID	Size	Silhouette	Average Year	Label (LLR)	Representative Keywords
0	35	0.824	2022	vestibular migraine	vestibular migraine; benign paroxysmal positional vertigo; persistent postural-perceptual dizziness; risk factors; precipitant conditions | vestibular rehabilitation; vestibular neuritis; chronic subjective dizziness; cervical vertigo; s disease
1	33	0.71	2022	vestibular rehabilitation	vestibular rehabilitation; cognitive behavioral therapy; body awareness; focus group; long-lasting dizziness | persistent postural-perceptual dizziness; cerebellar stroke; central vestibular dysfunction; multiple sclerosis; long-lasting dizziness
2	31	0.826	2023	functional dizziness	persistent postural-perceptual dizziness; functional dizziness; bilateral vestibulopathy; 3d spatial pointing task; spatial orientation | spatial navigation; spatial cognition; functional neurological disorder; chronic vertigo; vestibular disorders
3	30	0.721	2021	perceptual dizziness	persistent postural-perceptual dizziness; vestibular evoked myogenic potentials; romberg ratio; isolated otolith dysfunction; dizziness handicap inventory | chronic dizziness; anterior cingulate cortex; functional magnetic resonance imaging; persistent postural perceptual dizziness; angular gyrus
4	28	0.672	2022	dizziness handicap inventory	dizziness handicap inventory; beck depression inventory; vertiginous patients; balance deficit; risk assessment | persistent postural-perceptual dizziness; postural control; balance deficit; risk assessment; online support groups
5	17	0.843	2022	persistent postural-perceptual dizziness	persistent postural-perceptual dizziness; refractory dizziness; virtual reality; binary logistic regression analysis; kampo medicine | binary logistic regression analysis; vestibular system dysfunction; clinico-radiological characteristics; persistent posture-perceptual dizziness; potential risk factor
6	13	0.816	2022	acupuncture	persistent postural perceptual dizziness; hospital anxiety; menieres disease; depression scale; dizziness handicap inventory | functional neurological disorder; functional cognitive disorder; functional seizures; functional movement disorder; epileptic seizures

Note: Size: number of articles in each cluster; Silhouette: the average contour value of clustering (S > 0.7 indicates the clustering is highly convincing); LLR: Log-likelihood ratio.

**Table A3:** The most trafficked keyword for the top five modules each year.

Year	1990	1991	1992	1993	1994	1995	1996	1997	1998	1999
**Total modules**	-	-	-	-	7	7	9	9	11	9
**module1**	-	-	-	-	psychogenic_disorders	-	-	psychiatric_comorbidity	-	power_spectrum
**module2**	-	-	-	-	panic_disorder	-	-	panic_disorder	-	body_sway
**module3**	-	-	-	-	-	-	-	-	-	psychogenic_vertigo
**module4**	-	-	-	-	-	-	-	-	-	-
**module5**	-	-	-	-	-	-	-	-	-	-
**Year**	**200** **0**	**200** **1**	**200** **2**	**200** **3**	**200** **4**	**200** **5**	**20** **06**	**20** **07**	**20** **08**	**20** **09**
**Total modules**	7	6	4	6	7	6	9	9	11	9
**module1**	phobic_postural_vertigo	hypothesis	strategy	performance	chronic_vertigo	vestibular_function	panic_disorder	manual_medicine	central_causes	myogenic_potentials
**module2**	antagonist_muscles	anxiety	antagonist_muscles	panic_disorder	migraine	de_debarquement_syndrome	rehabilitation	agoraphobia	animal_models	follow_up
**module3**	-	phobic_postural_vertigo	visual_motion_stimulation	-	-	dysfunction	balance_control	disorders	downbeat_nystagmus	-
**module4**	-	-	-	-	-	menieres_disease	quantification	-	anxiety	-
**module5**	-	-	-	-	-	community_sample	-	-	-	-
**Year**	**201** **0**	**201** **1**	**201** **2**	**201** **3**	**201** **4**	**201** **5**	**20** **16**	**20** **17**	**20** **18**	**20** **19**
**Total modules**	8	13	10	12	11	12	13	14	9	11
**module1**	cognitive_behavioral_therapy	dizziness_handicap	diagnosis	control_mechanisms	symptoms	prevalence	compensation	cognitive_behavior_therapy	personality_traits	connectivity
**module2**	disorders	term_follow_up	chronic_subjective_dizziness	chronic_subjective_dizziness	dorsal_raphe_nucleus	functional_connectivity	chronic_dizziness	dorsal_cochlear_nucleus	cognitive_behavioral_therapy	depression
**module3**	dysfunction	cognitive_behavioral_treatment	depression_anxiety	german_version	-	-	agoraphobia	vestibular_neuritis	randomized_controlled_trial	bilateral_vestibulopathy
**module4**	chronic_dizziness	differential_diagnosis	-	dorsal_raphe_nucleus	-	-	phobic_postural_vertigo	downbeat_nystagmus	persistent_postural_perceptual_dizziness	diagnostic_criteria
**module5**	-	compression	-	vestibular_rehabilitation	-	-	-	vestibular_rehabilitation	committee	cognitive_behavioral_therapy
**Year**	**2020**	**2021**	**2022**	**2023**	**2024**	**2025**				
**Total modules**	15	16	13	14	12	8				
**module1**	cognitive_dysfunction	personality_traits	dizziness_handicap_inventory	vestibular_migraine	classification	consensus_document				
**module2**	chronic_subjective_dizziness	paroxysmal_positional_vertigo	sleep_quality_index	anxiety	persistent_postural-perceptual_dizziness	cognitive_behavioral_therapy				
**module3**	visual_vertigo	rehabilitation	postural_perceptual_dizziness	functional_cognitive_disorder	normative_values	blood_flow				
**module4**	vestibular_rehabilitation	chronic_vestibular_syndromes	physical_therapy	benign_paroxysmal_positional_vertigo	quality_of_life	sample				
**module5**	connectivity	canal_occlusion	binary_logistic_regression_analysis	residual_dizziness	postural_perceptual_dizziness	recovery				
